# 碳纳米管复合微球材料结合固相微萃取-气相色谱-串联质谱法检测环境水样中痕量多氯联苯

**DOI:** 10.3724/SP.J.1123.2025.01004

**Published:** 2025-10-08

**Authors:** Xinli SONG, Shuqi ZHU, Yuxin WU, Liyan ZHONG, Yuqing LIU

**Affiliations:** 枣庄学院食品科学与制药工程学院，山东 枣庄 277160; College of Food Science and Pharmaceutical Engineering，Zaozhuang University，Zaozhuang 277160，China

**Keywords:** 碳纳米管复合微球材料, 固相微萃取, 气相色谱-串联质谱, 多氯联苯, 环境水样, carbon nanotube composite microspheres materials, solid-phase microextraction （SPME）, gas chromatography-tandem mass spectrometry （GC-MS/MS）, polychlorinated biphenyls （PCBs）, environmental water sample

## Abstract

多氯联苯（PCBs）是目前国际上关注的常见持久性有机污染物之一，对自然环境和人类健康具有极大危害。本研究以碳纳米管复合微球（MWCNT@PS）为固相微萃取法（SPME）涂层材料，与气相色谱-串联质谱法（GC-MS/MS）相结合，建立了一种检测环境水样中痕量PCBs的分析方法。该涂层材料拥有良好的稳定性，对PCBs具有高效的萃取效果。本研究选择6种多氯联苯作为目标分析物，采用单因素优化法对影响萃取效果的重要因素进行了优化，获得了最优萃取条件（吸附时间为50 min，搅拌速率为600 r/min，pH值为6，NaCl浓度为1.5 mol/L，解吸温度为280 ℃，解吸时间为4 min）。采用气相色谱-三重四极杆质谱联用技术对水样品中多氯联苯进行定量分析，样品经TG-5 SILMS色谱柱（30 m×0.25 mm×0.25 μm）分离，采用电子轰击离子源和质谱多反应监测模式，实现了环境水样中6种多氯联苯的快速定性和定量分析。在优化条件下，所建立的分析方法具有线性范围宽（0.03~1 000 ng/L）、检出限低（0.01~0.03 ng/L）和重复性良好（相对标准偏差RSD≤9.38%，*n*=3）等优点。选择2 ng/L 的6种多氯联苯进行重复性实验，日内和日间RSD为1.64%~8.16%和2.83%~8.41%。将方法成功应用于桶装饮用水、雨水和河水3种实际环境水样中多氯联苯的检测，在低、中、高3个水平下，6种多氯联苯的加标回收率为82.4%~113.2%，实验结果表明，所建立的分析方法适用于环境中PCBs的富集与检测。本方法成功应用于实际环境水样中多氯联苯的定量分析，为快速、灵敏地检测环境水样中痕量多氯联苯提供了良好的思路。

## Abstract (en)

Polychlorinated biphenyls （PCBs） pose serious threats to the environment and human health because they are among the most common and persistent organic pollutants globally. In this study， six PCBs were extracted from environmental water samples using multiwalled carbon nanotubes （MWCNTs） on polystyrene （PS） microspheres as the solid-phase microextraction （SPME） coating material for gas chromatography-tandem mass spectrometry （GC-MS/MS）. This coating material is highly stable and exhibited a high extraction efficiency. 2，2′，5，5′-Tetrachlorobiphenyl （PCB-52）， 2，2′，4，5，5′-pentachlorobiphenyl （PCB-101）， 2，3′，4， 4′，5-pentachlorobiphenyl （PCB-118）， 2，2′，3，4，4′，5′-hexachlorobiphenyl （PCB-138）， 2，2′，4，4′，5，5′-hexachlorobiphenyl （PCB-153） and 2，2′，3，4，4′，5，5′-heptachlorobiphenyl （PCB-180） were chosen as target analytes. The main extraction factors were optimized using a single-factor optimization method， which led to the following optimum extraction conditions： adsorption time， 50 min； agitation speed， 600 r/min； pH， 6； NaCl concentration， 1.5 mol/L； desorption temperature， 280 °C； and desorption time， 4 min. GC coupled with triple quadrupole mass spectrometry was used to quantify PCBs in water samples. The chromatographic separation system was equipped with a TG-5 SILMS column （30 m×0.25 mm×0.25 μm）， with electron impact ionization and multi-reaction monitoring modes used during mass spectrometry. Five batches of MWCNT@PS were used as SPME coating materials， which were determined to have relative standard deviations （RSD） of less than 8.8% for the six PCBs. The reusability of the MWCNT@PS coating was also investigated； RSD of the recoveries of 2.8%–9.3% were obtained after ten SPME cycles with the same coating. These results reveal that the MWCNT@PS coating materials are highly stable and reusable in SPME applications， with good results obtained under the optimal conditions. The established method exhibited linearity in the range of 0.03–1 000 ng/L for PCB-52 and PCB101， 0.07–1 000 ng/L for PCB118， PCB138， and PCB 153， and 0.10–1 000 ng/L for PCB-180， with correlation coefficients of 0.993-0.998. Limits of detection and quantification （LODs and LOQs， respectively） of 0.01–0.03 and 0.03–0.10 ng/L were determined for the developed method. The intra- and inter-day precisions exhibited RSDs of 1.64%–8.16% and 2.83%–8.41%， respectively， at 2 ng/L， 3.31%–7.19% and 3.79%–9.12%， respectively， at 10 ng/L， and 2.70%–9.38% and 4.04%–8.56%， respectively， at 100 ng/L. The established method was used to determine six PCBs in barreled drinking water， rainwater， and three environmental river water samples. No PCBs were found in barreled drinking water， rain water and river water. Satisfactory recoveries of 82.4%-113.2% were achieved at low， medium， and high levels. Accordingly， the MWCNT@PS-composite-coating-material-based SPME-GC-MS/MS method is accurate and effective. This study revealed that MWCNT@PS composites provide a good avenue for the rapid and sensitive detection of PCBs in water samples.

多氯联苯（PCBs）是目前国际上关注的常见持久性有机污染物之一，毒性强，具有良好的耐热性和高化学稳定性，难以降解，且该类污染物易致突变、致癌、致畸，对自然环境和人类健康具有极大危害，受到国内外的广泛关注^［1，2］^。PCBs曾作为20世纪优质工业添加剂广泛应用于塑料、化工和印刷等行业，尽管已经停止使用多年，但由于其持久性和稳定性导致其仍在环境中残留且积累，主要分布在水、土壤和沉积物等物质中^［3-5］^。所以，需要建立一种准确、快速、有效的方法来检测环境中的PCBs。

由于PCBs在环境水样中的痕量分布以及基质效应的干扰，在定量分析时，样品前处理过程必不可少。固相微萃取（SPME）技术是一种集富集和进样于一体的样品前处理技术，该方法因具有不消耗有机溶剂、萃取材料用量少、易于仪器联用和重复性好等优点，在样品前处理领域广泛应用^［6，7］^。在该技术中，目标分析物在纤维涂层和溶液中分配平衡，所以，优异萃取性能的涂层材料在SPME技术中异常重要。商品化固相微萃取纤维存在价格昂贵、易损坏、寿命短和易膨胀等缺点。因此发展新的萃取纤维材料成为这一研究领域的热点。目前，已有共价有机框架^［8-10］^、纳米花^［11］^、空心纳米笼^［12］^、聚苯乙烯纳米球^［13］^等多种新型复合材料作为SPME的涂层材料，成功应用于环境中PCBs的分析检测。

多壁碳纳米管（MWCNT）化学性质稳定，表面积高和吸附能力强，是一种优良的吸附材料^［14，15］^。MWCNT在水溶液中容易团聚，影响了和目标物的接触面积，进而影响萃取效率。因此在MWCNT中引入聚苯乙烯微球作为模板，通过层层自主装技术，MWCNT覆盖在聚苯乙烯微球的表面，形成以MWCNT为壳的核壳结构的复合微球材料，这样既保留了MWCNT的强吸附能力又增加了MWCNT和目标分析物的接触面积，提高了复合微球材料对目标分析物的萃取效果^［16］^。聚苯乙烯微球作为模板易于合成，稳定性好，操作简单^［17］^。本研究利用MWCNT@聚苯乙烯微球（PS）作为SPME的涂层材料，结合GC-MS/MS对水样中最常见的6种PCBs进行萃取和定量。采用单因素实验对影响萃取效率的主要因素进行了评估和优化，建立了一种用于环境水样中痕量PCBs的灵敏度高、准确度好、重复性强的检测分析方法。

## 1 实验部分

### 1.1 仪器、试剂与材料

气相色谱-三重四极杆质谱仪（TSQ9610，美国Thermo Scientific公司）。扫描电子显微镜（JSM7800F，日本JEOL公司）。热重分析仪（STA409PC，德国Netzsch公司）。

聚（二烯丙基二甲基氯化铵）（PDDA，*M*_r_ 400 000）和聚（4-苯乙烯磺酸钠）（PSS，*M*_r_ 70 000）购自上海阿拉丁试剂有限公司。PS（直径约为3 μm）购自苏州纳微科技股份有限公司。羧基化多壁碳纳米管购自南京先丰纳米材料科技有限公司（长度0.5~2 μm，直径20~30 nm）。2，2′，5，5′-四氯联苯（PCB-52）、2，2′，4，5，5′-五氯联苯（PCB-101）、2，3′，4，4′，5-五氯联苯（PCB-118）、2，2′，3，4，4′，5′-六氯联苯（PCB-138）、2，2′，4，4′，5，5′-六氯联苯（PCB-153）和2，2′，3，4，4′，5，5′-七氯联苯（PCB-180）均为色谱纯，购自上海阿拉丁试剂有限公司。桶装水购于山东国新力源水业有限公司，雨水和河水分别采集于枣庄学院和枣庄西沙河。

### 1.2 标准溶液的配制

6种多氯联苯混合标准溶液在4 ℃下保存。使用时用甲醇逐级稀释至所需浓度，现配现用。

### 1.3 MWCNT@PS的制备

称取50 mg PS微球加入到40 mL PDDA水溶液中（3 mg/mL）搅拌3 h，重复离心分离（3 000 r/min，3 min）和水洗3次；接着将吸附有PDDA的PS加入到40 mL PSS水溶液中（3 mg/mL）搅拌3 h，离心分离（3 000 r/min，3 min）和水洗3次后；接着再重复进行一次PDDA的吸附操作，这样在PS微球表面形成了PDDA/PSS/PDDA的3层聚电解质。把吸附有3层聚电解质的PS微球加入40 mL MWCNT水溶液（1 mg/mL）中，搅拌40 min后，离心分离（3 000 r/min，3 min）和水洗3次。接着交替进行PDDA和MWCNT的吸附组装，重复以上操作5次，就可以得到MWCNT@PS表面组装多层碳纳米管的复合微球材料。

### 1.4 固相微萃取装置的制备

将不锈钢丝一端3 cm置于氢氟酸中腐蚀4 h，然后用纯水超声清洗。在变细的部分表面均匀涂上硅酮密封胶，然后在胶的表面黏附上MWCNT@PS涂层材料。将带有涂层的不锈钢丝装入5 μL气相色谱进样针中，即得自制固相微萃取装置。

### 1.5 SPME过程

环境水样用0.22 µm滤膜过滤后存储在棕色玻璃瓶中，于4 ℃冰箱中避光保存。萃取模式为浸入式。将10 mL环境水样置于20 mL玻璃顶空瓶中，使用NaCl调节溶液盐浓度至1.5 mol/L，用盐酸或氢氧化钠调节pH值至6，将自制的固相微萃取针头扎入瓶中，在水中振荡萃取50 min，搅拌速率为600 r/min。从顶空瓶中拔出后放入气相色谱进样口中解吸4 min，解吸温度为280 ℃，进行GC-MS/MS分析。

### 1.6 气相色谱-质谱条件

色谱柱TG-5 SILMS（30 m×0.25 mm×0.25 μm，美国Thermo Scientific公司）。初始温度为150 ℃保持1 min，以10 ℃/min升至290 ℃，并保持4 min。进样口温度为280 ℃。

四极杆温度为150 ℃。载气为氦气（99.999%），流速为1.0 mL/min。采用多反应监测（MRM）模式。离子源为EI源，温度为250 ℃。其他参数见表1。

**表1:** 6种PCBs的保留时间和质谱参数

Compound	Retention time /min	Quantitive ion pair		Qualitative ion pair	
		Collision energy/eV	Monitored transition （*m/z*）	Collision energy/eV	Monitored transition （*m/z*）
2，2′，5，5′-Tetrachlorobiphenyl （PCB-52）	8.7	34	292.0/220.0	34	292.0/222.0
2，2′，4，5，5′-Pentachlorobiphenyl （PCB-101）	10.3	34	326.0/256.0	34	324.0/254.0
2，3′，4，4′，5-Pentachlorobiphenyl （PCB-118）	11.5	32	324.0/254.0	32	326.0/256.0
2，2′，3，4，4′，5′-Hexachlorobiphenyl （PCB-138）	11.9	20	360.0/290.0	20	358.0/288.0
2，2′，4，4′，5，5′-Hexachlorobiphenyl （PCB-153）	12.3	20	358.0/288.0	20	360.0/290.0
2，2′，3，4，4′，5，5′-Heptachlorobiphenyl （PCB-180）	13.6	36	394.0/324.0	36	396.0/326.0

## 2 结果与讨论

### 2.1 MWCNT@PS的表征

MWCNT@PS复合微球材料的扫描电镜如图1a所示。因为MWCNT本身的强吸附性和柔韧性保证了它能够经过层层组装后，可以沉积在PS微球表面上。组装的第一步先用聚电解质处理PS微球，用PDDA和PSS交替吸附在微球表面，形成3层聚电解质修饰的微球表面，有助于后面MWCNT吸附在微球表面。开始组装后，PS微球表面由相对光滑变成了相对粗糙，微球表面一些毛茸茸的线状物，这些线状物就是碳纳米管，随着自组装层数的增加，组装到微球表面的碳纳米管越来越多，粗糙程度也逐渐增加，碳纳米管相互缠绕，紧紧包覆在微球表面。组装了5层PDDA/MWCNT后，MWCNT已经完全均匀地包裹在PS微球表面，形成了相对致密和厚度可控的MWCNT外壳（图1a）。如图1b和1c所示，在自制的不锈钢纤维表面上有形貌均匀的MWCNT@PS涂层，该涂层由许多微球堆积而成，结果表明成功制备了MWCNT@PS复合微球，并制成了均匀的SPME涂层。该涂层的厚度约为30 μm。

**图1:**
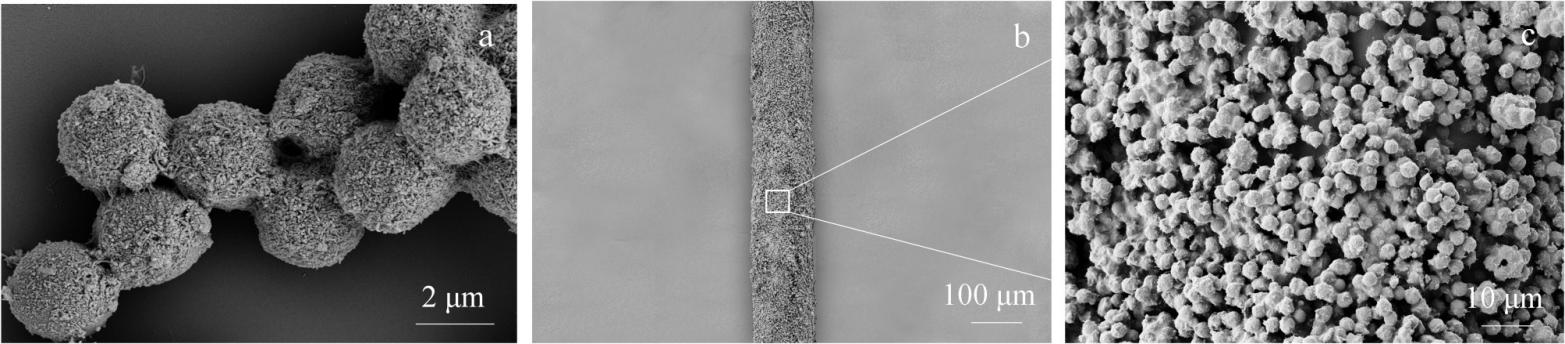
（a）MWCNT@PS的扫描电子显微镜图、MWCNT@PS涂层纤维在（b）低倍镜下和（c）高倍镜下的扫描电子显微镜图

### 2.2 MWCNT@PS的热稳定性

实验中将MWCNT@PS作为SPME涂层材料，需要对其热稳定性进行考察。在氮气保护下，升温速度为10 ℃/min。结果表明，当温度加热至300 ℃时，MWCNT@PS的质量损失约为5%，说明MWCNT@PS材料具有优良的热稳定性，能够满足气相进样口高温解吸。因此，MWCNT@PS可作为SPME涂层材料。

### 2.3 SPME条件的优化

为了获得最优的实验条件，实验分析了6种PCBs混合标准溶液（1 ng/L），以化合物的峰面积为指标，通过单因素实验，对萃取时间、搅拌速率、pH值、离子强度、解吸温度和解吸时间6个影响因素进行了考察。

#### 2.3.1 萃取时间

实验对萃取时间在15~60 min内的萃取效果进行了优化。如图2a所示，在15~50 min范围内，萃取效率随着时间的延长而增加，当时间为50 min时，达到了萃取平衡。富集50 min后萃取效率维持在稳定的水平，表明已接近饱和状态。从时间效率和灵敏度效果考虑，选择萃取时间为50 min。

**图2:**
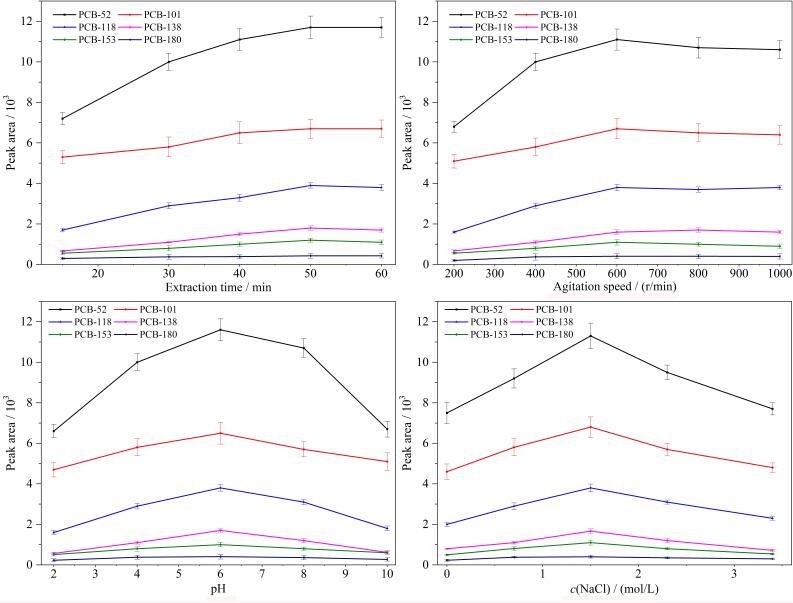
（a）萃取时间、（b）搅拌速率、（c）样品pH值和（d）离子强度对萃取效果的影响（*n*=3）

#### 2.3.2 搅拌速率

考察了搅拌速率（200~1 000 r/min）对萃取效率的影响。图2b结果表明，在200~600 r/min范围内，所有萃取效率都随着搅拌速率的增加而增加，这是由于搅拌有利于目标物向涂层的迁移和扩散，从而提高萃取效率。在600 r/min时达到最大值。在600~1 000 r/min范围内，萃取效率没有明显改变。因此，选择搅拌速率为600 r/min，用于之后的实验研究。

#### 2.3.3 萃取效率

考察了pH 2~10对萃取效率的影响。如图2c所示，PCBs在不同酸性下，目标分析物在吸附涂层和溶液中的分配系数不同，萃取效果发生变化。萃取效率在pH为6时达到最佳水平，因此实验选择最优pH为6。

#### 2.3.4 离子强度

考察了离子强度对萃取效果的影响。调整NaCl浓度（0~3.4 mol/L）以实现离子强度的改变。如图2d所示，当NaCl浓度增加至1.5 mol/L时，萃取效果提高。当NaCl浓度从1.5 mol/L升至3.4 mol/L时，萃取效率呈下降趋势，盐浓度过高，萃取溶液的黏度和密度增大，阻碍了目标物的扩散，不利于目标物的萃取。本研究选择NaCl浓度为1.5 mol/L。

#### 2.3.5 解吸温度

对解吸温度在220~320 ℃范围内进行了研究，如图3a所示，随着解吸温度的升高，萃取效率增加，当解吸温度为280 ℃时，对PCBs的萃取效果达到最佳，目标分析物完全从涂层中解吸。解吸温度过高可能会降低纤维涂层的使用寿命，因此，选择解吸温度为280 ℃。

**图3:**
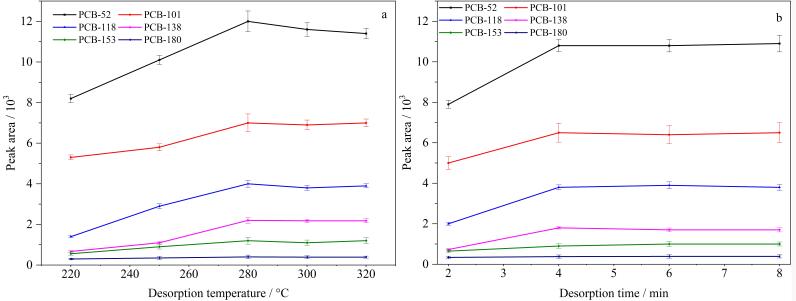
（a）解吸温度、（b）解吸时间对萃取效果的影响（*n*=3）

#### 2.3.6 解吸时间

实验还考察了解吸时间在2~8 min范围内对萃取效果的影响，随着解吸时间的延长，萃取效果上升，当解吸时间为4 min时，6种PCBs的萃取效率达到最佳（图3b）。继续增加解吸时间，萃取效果基本不变。因此，后续实验的解吸时间选为4 min。

在最佳条件下，MWCNT@PS与MWCNT、单层碳纳米管组装的微球材料（PDDA/MWCNT）_1_对PCBs萃取效果进行了比较，MWCNT@PS对PCBs的萃取效率最高。MWCNT@PS比MWCNT对PCBs的萃取效率高1.4~1.9倍（图4），证明了该碳纳米管复合微球材料对PCBs萃取的灵敏性和高效性。

**图4:**
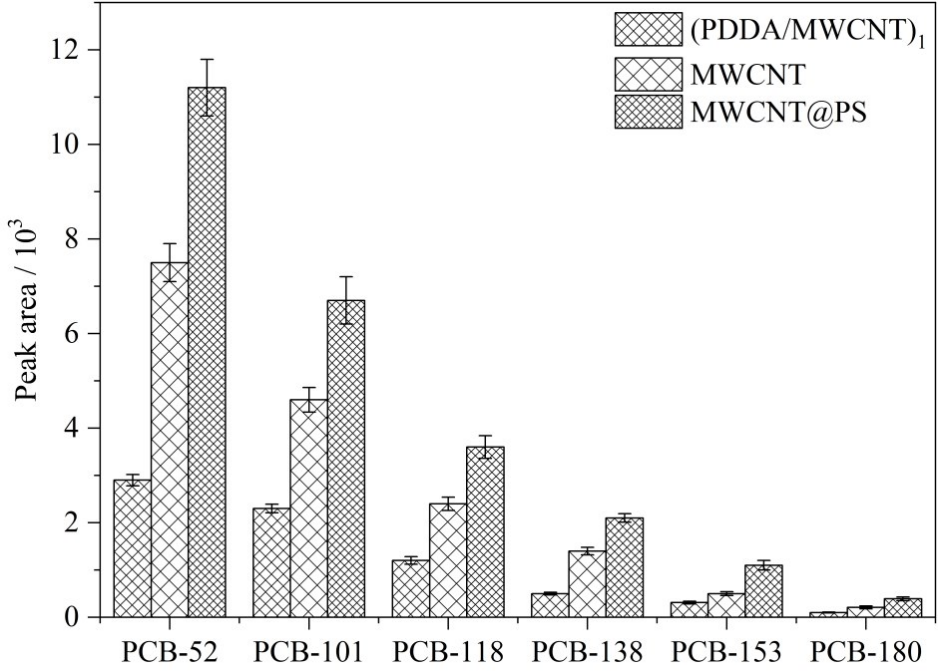
（PDDA/MWCNT）_1_、MWCNT与MWCNT@PS 3种材料对PCBs的萃取效率（*n*=3）

### 2.4 方法学参数

在上述最优的萃取条件下，对建立的方法进行了考察。PCB-52和PCB-101的线性范围为0.03~1 000 ng/L，PCB-118、PCB-138和PCB-153的线性范围为0.07~1 000 ng/L，PCB-180的线性范围为0.10~1 000 ng/L，6种PCBs在其线性范围内线性良好，相关系数（*r*）为0.993~0.998。该分析方法的检出限（LOD，*S/N*=3）为0.01~0.03 ng/L，定量限（LOQ，*S/N*=10）为0.03~0.10 ng/L（表2）。

**表2:** 6种PCBs的线性范围、相关系数、检出限和定量限

Compound	Linear range/（ng/L）	*r*	LOD/（ng/L）	LOQ/（ng/L）
PCB-52	0.03-1000	0.998	0.01	0.03
PCB-101	0.03-1000	0.996	0.01	0.03
PCB-118	0.07-1000	0.996	0.02	0.07
PCB-138	0.07-1000	0.995	0.02	0.07
PCB-153	0.07-1000	0.995	0.02	0.07
PCB-180	0.10-1000	0.993	0.03	0.10

在2、10和100 ng/L 3个水平下对6种PCBs进行重复性试验，日内（日间）的相对标准偏差（RSD）≤9.38%（*n*=3）（表3），表明了该分析方法稳定。以5个不同批次间制备的材料作为吸附剂进行考察，目标物萃取效率的RSD为3.2%~8.8%，结果表明MWCNT@PS具有良好的制备重复性。同一根萃取涂层材料连续使用10次后，目标物萃取效率的RSD为2.8%~9.3%，表明MWCNT@PS具有较好的可重复使用性。在最佳萃取条件下，单根涂层纤维使用60次后，对PCBs的萃取效果在80%以上。

**表3:** 6种PCBs的日内精密度和日间精密度（*n*=3）

Compound	2 ng/L		10 ng/L		100 ng/L	
	Intra-day RSD/%	Inter-day RSD/%	Intra-day RSD/%	Inter-day RSD/%	Intra-day RSD/%	Inter-day RSD/%
PCB-52	3.45	2.83	4.28	5.20	4.28	5.81
PCB-101	1.64	2.91	7.03	9.12	2.70	4.04
PCB-118	4.80	8.41	7.19	7.93	3.89	8.56
PCB-138	5.12	4.72	3.31	3.79	6.19	5.29
PCB-153	8.16	7.62	5.02	6.39	5.90	8.01
PCB-180	7.94	6.21	3.92	4.81	9.38	7.31

与其他文献报道的检测PCBs分析方法进行比较，该方法具有更低的检出限和更宽的线性范围（表4）。尽管分散固相萃取（dSPE）和磁固相萃取（MSPE）方法萃取时间较短^［20，21］^，但本方法不消耗溶剂且解吸过程更为简单。和表4中吸附材料比较，本方法使用的吸附材料MWCNT@PS，制备简单，易于操作，成本低廉。

**表4:** 本方法与其他报道方法的比较

Adsorption material	Extraction method	Analytical method	Linear range /（ng/L）	LOD/（ng/L）	Extraction time/min	Samples
MIL-on-UiO ^［11］^	SPME	GC-FID	1-50000	0.1-2	30	water， orange juice
Co_3_N_4_@C_3_N_5_^［18］^	SPME	GC-FID	0.5-20000	0.17-61	40	water
COF TFPB-BD ^［19］^	SPME	GC-MS/MS	1-1000	0.08-0.35	50	aquatic products
Pd-MONT ^［20］^	d-SPE	GC-MS/MS	2-1000	0.26-0.82	4.5	water
Fe_3_O_4_@MOF ^［21］^	MSPE	GC-MS/MS	5-4000	1.1-1.6	32	water
MWCNT@PS （this method）	SPME	GC-MS/MS	0.03-1000	0.01-0.03	50	water

d-SPE： dispersive solid phase extraction； MSPE： magnetic solid phase extraction.

选用了30 μm PDMS和50/30 μm DVB/CAR/PDMS两种商品化涂层进行萃取效果比较。在优化的实验条件下，萃取水样中1 ng/L 的PCBs目标分析物，结果显示，自制MWCNTs@PS涂层的萃取效率明显高于两根商品化涂层（图5）。

**图5:**
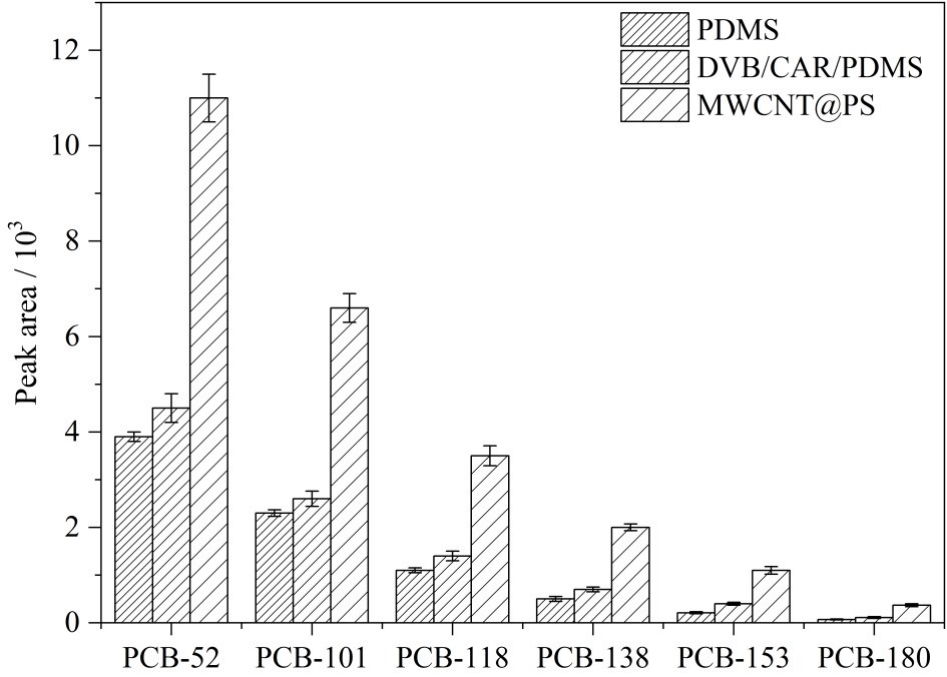
碳纳米管复合微球涂层与商品萃取涂层萃取效率对比

### 2.5 实际水样中PCBs的分析

为了评价本方法的适用性，选择3种环境水样（桶装饮用水、雨水和河水），采用建立的SPME-GC-MS/MS方法进行检测。结果表明，桶装饮用水、雨水和河水中均没有检测到6种PCBs。在3种环境水样中添加3个水平的PCBs混合标准溶液（2、10和100 ng/L）对方法的准确度和精密度进行研究，如表5所示，该方法的加标回收率为82.4%~113.2%。图6为桶装饮用水加标样品的色谱图。

**表5:** 环境水样中6种PCBs的加标回收率（*n*=3）

Compound	Added level/（ng/L）	Recoveries/%		
		Rain water	Barreled drinking water	River water
PCB-52	2	83.1±2.7	85.4±4.2	110.3±8.7
	10	87.5±3.9	82.4±6.1	92.1±4.3
	100	90.4±4.3	107.3±4.6	98.2±9.1
PCB-101	2	106.3±5.5	94.6±5.0	89.2±5.3
	10	87.3±4.7	87.1±2.7	90.1±2.8
	100	94.2±2.8	92.6±8.4	82.9±5.4
PCB-118	2	97.4±7.2	86.6±3.6	94.8±7.7
	10	87.3±5.0	109.3±7.9	102.4±9.2
	100	90.3±7.8	97.3±4.1	87.9±4.4
PCB-138	2	89.3±3.0	98.1±8.8	113.2±5.8
	10	104.7±9.4	84.0±2.8	93.8±7.6
	100	97.3±2.4	95.3±3.5	99.1±4.3
PCB-153	2	92.6±3.8	106.1±7.3	90.2±7.1
	10	87.2±7.3	98.3±6.8	96.4±4.6
	100	85.0±2.2	96.2±6.2	91.7±8.4
PCB-180	2	98.4±9.2	91.2±8.3	103.2±7.9
	10	108.4±6.3	89.2±4.5	98.1±5.3
	100	93.6±3.2	100.6±5.8	92.7±3.3

**图6:**
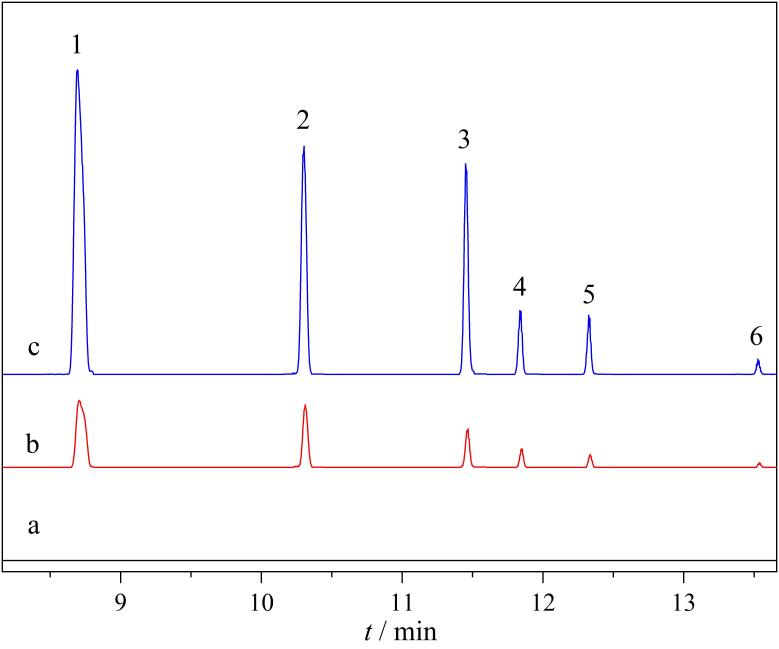
（a）空白桶装水、加标（b）2 ng/L和（c）10 ng/L PCBs后的色谱图 1. PCB-52； 2. PCB-101； 3. PCB-118； 4. PCB-138； 5. PCB-153； 6. PCB-180.

## 3 结论

本研究制备了一种核壳碳纳米管微球材料MWCNT@PS，并将其作为SPME涂层材料，与GC-MS/MS结合，建立了一种简单、灵敏的检测环境水样中痕量多氯联苯的分析方法。该涂层材料拥有良好的热稳定性和循环使用性。在最佳的萃取条件下，该方法具有检出限低、线性范围宽、萃取效率高等优点。最后，本分析方法成功应用于环境水样中痕量多氯联苯的分析，取得了满意的结果，为环境水样中痕量PCBs的检测提供了一种新思路。
